# A Versatile qPCR for Diagnosis of Leporid Gammaherpesvirus 5 Using Evagreen^®^ or Taqman^®^ Technologies

**DOI:** 10.3390/v13040715

**Published:** 2021-04-20

**Authors:** Fábio A. Abade dos Santos, Carina L. Carvalho, Maria C. Peleteiro, Francisco Parra, Margarida D. Duarte

**Affiliations:** 1 Instituto Nacional de Investigação Agrária e Veterinária, Av. da República, Quinta do Marquês, 2780-157 Oeiras, Portugal; carina.carvalho@iniav.pt (C.L.C.); margarida.duarte@iniav.pt (M.D.D.); 2 CIISA, Faculdade de Medicina Veterinária, Universidade de Lisboa, Avenida da Universidade Técnica, 1300-477 Lisboa, Portugal; mcpelet@fmv.ulisboa.pt; 3 Instituto Universitario de Biotecnología de Asturias (IUBA), Departamento de Bioquímica y Biología Molecular, Universidad de Oviedo, 33006 Oviedo, Spain; fparra@uniovi.es

**Keywords:** herpesvirus, gammaherpesvirus, leporid gammaherpesvirus 5, *Lepus granatensis*, Iberian hare, reproduction, qPCR

## Abstract

In late 2019, the first herpesvirus in the genus Lepus, named leporid gammaherpesvirus 5 (LeHV-5) was described. At the time, herpetic typical lesions were observed in hares infected by the myxoma virus, which is known to induce immunosuppression. Though the real impact of LeHV-5 is still poorly understood, since it affects reproduction, it poses an additional threat to the already fragile populations of Iberian hare, demanding prevalence investigations. In this article, we describe the first quantitative molecular method for LeHV-5 detection, using either Taqman or the EvaGreen systems. This method has excellent sensitivity and specificity, it is able to detect 2.1 copies of LeHV-5 DNA and was validated with an internal control targeting the 18S rRNA gene, allowing monitoring extraction and PCR amplification efficiencies.

## 1. Introduction

The Iberian hare (*Lepus granatensis*) solely inhabits the Iberian Peninsula and is currently considered a “least concern“ species by the International Union for Conservation of Nature, although a declining trend was recognized in the 2019 review [1,2]. The Iberian hare is the only hare species found in Portugal and in the last years, many local populations became extinct or significantly reduced due to cumulative changes in the habitat and emergence of new pathogens. Multidisciplinary approaches to investigate the causes of mortality and decline of the Iberian hare and European rabbit (*Oryctolagus cuniculus*), both key wild species of the Mediterranean ecosystems, is paramount for biodiversity conservation [1,3].

There are no studies evaluating the different causes of the decline of the Iberian hare populations, and the health status of this species is less well-known than that of rabbits. Recently, researchers became more interested in the Iberian hare after the emergence of myxomatosis [4,5] in 2018. Until then, myxomatosis only affected rabbits, and as reported, other species of hares [6,7,8].

Very recently, leporid gammaherpesvirus 5 (LeHV-5), the first herpesvirus found in hares, was described [9], but its real impact on the population is still difficult to evaluate or anticipate. However, the occurrence of genitalia necrosis, one of the lesions associated with LeHV-5 infection, shows its inevitable impact on reproductive activity and birth rate. LeHV-5 was also detected in apparently healthy hares, suggesting that LeHV-5 is circulating in the population, as observed with other human gammaherpesvirus (e.g., Epstein-Barr virus). Additionally, the high number of symptomatic co-infections by LeHV-5 and the recombinant myxoma virus (ha-MYXV) [1,9] suggests that immunosuppression caused by MYXV triggers the necessary mechanisms for reactivation of LeHV-5 in asymptomatic infected hares. Given the fragile state of Iberian hare populations in Portugal and Spain after mid-2018, and since ha-MYXV is endemic in the Iberian Peninsula, it is important to investigate the LeHV-5 geographic spread in wild populations, and to assess the overlap with areas where MYXV epidemics are recurrent.

To improve the detection of LeHV-5, a real-time PCR system targeting the LeHV-5 glycoprotein B encoding gene was developed, validated, and optimized, to be used by TaqMan or EvaGreen methods.

To avoid false results, we normalized the qPCR method to detect failures in the extraction process and amplification reaction due to inhibitor factors. The internal reference chosen was the 18S rRNA housekeeping gene, widely described as a reliable normalization gene [10]. The method proved to be a highly sensitive and specific tool to diagnose LeHV-5 in a single analysis, through a fast and simple process.

Monitoring LeHV-5 and MYXV would allow us to understand their mutual inference and to elucidate the pathophysiology of the two infections.

## 2. Materials and Methods

### 2.1. Biological Samples

No animals were scarified for this study as only hares found dead in the field were used. Carcasses arrived at the Portugal National Reference Laboratory (INIAV, I.P.) within the scope of the +Coelho Project (Dispatch no. 4757/2017 of 31 May). During the necropsies, samples of liver and spleen were collected for virology investigations and preserved at −20 °C, until analysis.

### 2.2. DNA Extraction

For nucleic acid extraction, fresh samples of liver and spleen were homogenised at 20% (*w/v*) with phosphate buffered saline (PBS) and clarified at 3000 g for 5 min. Total DNA and RNA were extracted from 200 μL of the clarified supernatants, using the MagAttract 96 cador Pathogen Kit (Qiagen, Hilden, Germany) in a BioSprint 96 nucleic acid extractor (Qiagen, Hilden, Germany), according to the manufacturer’s protocol. The nucleic acids were preserved at −20 °C until use.

### 2.3. Primer Design

The primers were designed based on LeHV-5 gB nucleotide sequences obtained previously (Abade dos Santos et al., 2020) (MN557129 to MN557133), using PCR Primer Design software (Eurofins Genomics, Ebersberg, Germany) but manually optimised. The nucleotide sequences of the primers and probe are described in Table 1. The target regions for the primers and probe were preserved in all samples analysed (*n* = 20, results not shown). The size of the amplicon generated was 200 bp.

To be included as a dual internal extraction and amplification control in the duplex qPCR, we designed two primers within the 18S ribosomal RNA gene, using a partial 18S rRNA gene sequence of *Lepus* europaeus (AY150540, GenBank). Primers were validated in silico and in vitro.

### 2.4. Cloning

The amplicons generated by the LeHV-5gB and 18S gene conventional PCR systems (Table 1) were visualized by agarose gel electrophoresis, and the reaction product was purified and directly cloned into the pCR2.1 TA vector (Invitrogen Corporation, San Diego, CA, USA), according to the manufacturer’s recommendations.

Transformation of the *Escherichia coli* competent cells (one-shot TOP 10, Invitrogen) was carried out as recommended. White colonies were isolated and inoculated in 5 mL of Luria Broth medium and grown overnight at 37 °C with 220 rotations per minute. Recombinant DNA was obtained using the NZYMiniprep Kit (NZYTech, Lisbon, Portugal). Three recombinant plasmids containing partial sequences from the LeHV-5 gB, 18S hare, and 18S rabbit were purified from the bacterial cultures.

Both DNA strands of the pgB, p18S_ha (*Lepus granatensis*) and p18S_r (*Oyctolagus cuniculus algirus*) inserts were sequenced using the M13F and M13R primers and a 3130 Genetic Analyser (Applied Biosystems, Foster City, CA, USA). The 117bp sequences from p18S_ha and p18S_r were aligned and used to design the probe (Table 1). Sequences were assembled and edited using the Bioedit 7.2 software (version?).

### 2.5. Evaluation of the PCR Component Variation

The LeHV-5 and 18S rDNA qPCR systems were first optimized separately by analysing the impact of variation of several parameters in the PCR performance. The main variables for qPCR amplification were crossed, to identify each optimal condition, namely the annealing temperature (52 to 62 °C), the dNTP concentration (0.2 mM to 0.4 mM), the MgCl_2_ final concentration (2.5 mM to 6 mM), and the primer concentration (0.1 and 0.3 µM). These combinations were tested using 10^8^ and 10 copies of pgB or p18S_ha. The NZYTaq II 2× Colourless Master Mix (NZYTech, Lisbon, Portugal) was used in these assays, adapting the dNTP and MgCl_2_ concentrations with standard solutions.

The final reaction volume was 25 μL, including 0.5 μL of each forward primer (variable final concentrations), 0.5 μL of each reverse primer (variable final concentrations), 0.5 μL of each probe (fixed 0.2 μM final concentration) and 5 μL of the DNA template. RNase/DNase free water was used to make up the final volume. The amplification was run on a CFX96 real-time system associated with C1000 thermal cycle (BIORAD) under the following conditions—initial denaturation at 95 °C for 3 min followed by 39 cycles of denaturation at 94 °C for 30 s; variable annealing temperature for 30 s and extension at 72 °C for 30 s. These combinations were also used for testing the interference between the two pairs of primers and the two probes, by analysing the effects in the Ct and Fluorescence values.

### 2.6. Field Samples Analysis

For field samples, the final reaction volume was 25 μL for the Taqman system, which comprised 12.50 μL of Multiplex PCR NZYTaq 2× Colourless Master Mix, 0.5 μL of each forward primer (0.2 μM final concentration), 0.5 μL of each reverse primer (0.2 μM final concentration), 0.5 μL of each probe (0.2 μM final concentration), 5 μL of DNA template from each sample, and 4.5 μL RNase/DNase free water. The amplification was run on a CFX96 real-time system associated with the C1000 thermal cycle (BIORAD) under the following conditions—initial denaturation at 95 °C for 3 min followed by 49 cycles of denaturation at 94 °C for 30 s, annealing at 62 °C for 30 s and extension at 72 °C for 30 s.

For the EvaGreen system, the final reaction volume was 20 μL comprising 10 μL of SsoFast™ EvaGreen^®^ Supermix, 0.5 μL of forward primer (0.2 μM final concentration), 0.5 μL of reverse primer (0.2 μM final concentration), 5 μL of DNA template from each sample, and 6.5 μL RNase/DNase free water. The amplification was run on a CFX96 real-time system associated with the C1000 thermal cycle (BIORAD), under the following conditions—initial denaturation at 98 °C for 2 min followed by 39 cycles of denaturation at 98 °C for 5 s, annealing and extension at 62 °C for 5 s. Finally, the melt curve was analyzed at a range of temperatures of 65–95 °C (0.5 °C increment), 5 s/step. In this case, the 18S internal control was not incorporated, in order to avoid confusing the result of the amplification curve. However, a High Resolution Melt Analysis allowed for its inclusion (results not shown).

### 2.7. Detection Limit, Sensitivity, Specificity, Repeatability, and Reproducibility of the qPCR

The specificity of the primers and probes of the LeHV-5 gB and 18S rDNA qPCR systems were first determined in silico using the BLASTN analysis service from NCBI. To further extend the specificity evaluation of the method, total genomes (or the glycoprotein B gene in case of some herpesviruses) of potential rabbit and hare pathogens were tested against the LeHV-5 primers and probe, with very relaxed temperature conditions (namely showing all matching sites of primer binding, a length of 60 bp to 3000 bp and allowing mismatches in 5 nucleotides of the 3′end), looking for all possible connection points using the software FASTPCR 6.7 (PrimerDigital, 2020).

The genomes tested included *Bibersteinia trehalosi* (NZ_CP006954), *Chlamydophila abortus* (CR848038), *Coxiella burnetii* (CP040059), *Cryptosporidium parvum* (CM000436), *Escherichia coli* (AE014075), *Encephalitozoon cuniculi* (LFTZ0100z0003), *Enterococcus faecalis* (CP045918), *Francisella tularensis* (CP025778), *Klebsiella pneumoniae* (FO203501), *Leptospira interrogans* (CP039256), *Mannheimia haemolytica* (CP006957), *Pasteurella multocida* (CP031552), *Salmonella enterica* (CP003278), *Serratia* sp. (CP025085), *Staphylococcus aureus* (AP017922), *Staphylococcus epidermidis* (CP043847), *Toxoplasma gondii* (U87145), *Myxoma virus* (KP723389 and KP723390), and *ha-MYXV* (MK340973), *and other species of herpesvirus that do not affect hares, including Macacine herpesvirus 5 (NC_003401), Human herpesvirus 2 (KU310663), Macaca mulatta rhadinovirus (AF210726), Gorilla rhadinovirus 1 (AY177144), Rodent herpesvirus (NC_015049), Apodemus agrarius rhadinovirus (AY854168), Mus cervicolor rhadinovirus 1 (DQ821582), Apodemus flavicollis rhadinovirus 1* (DQ821580), and Microtus agrestis rhadinovirus 1 (EF128052). Some RNA viruses that might not be amplified by this DNA-based qPCR system, were also evaluated given that they might be included in a multiplex RNA/DNA PCR System in the future. These included hare lagovirus (KR230102, MK138384), RHDV (MF421574), and the European brown hare syndrome virus (MK440616).

To assess the specificity of the method, the system was also tested in vitro (both as a uniplex and duplex) using samples of the total nucleic acid extracted from animal tissues infected with the following species of viruses, *bovine herpesvirus* (MCFV, BoHV-1 and BoHV-4), *Equine herpesvirus* (EHV-1, EHV-2 and EHV-5), *Canine herpesvirus* (CaHV-1), *Felid herpesvirus* (FeHV-1), *Gallid herpesvirus 1* (GaHV-1) and *2* (GaHV-2), *Psittacid herpesvirus 1* (PDV), myxoma virus (from rabbit and Iberian hare), rabbit haemorrhagic disease virus, total nucleic acid of rabbit, and hare isolated colonies of *E.coli, P. multocida,* and *S. aureus*, and total nucleic acid from the isolated parasites of *Cysticercus pisiformis*, *Eimeria stiedae*, and *Eimeria* spp. and *Passalurus ambiguous,* available at the National Reference Laboratory for Animal Health (INIAV, I.P, Oeiras, Portugal). In all reactions, 20 ng of the template was used. The PCRs were performed using the conditions described above in this manuscript, along with a more relaxed annealing temperature of 55 °C.

DNA concentrations of plasmid pgB, p18S_ha, and p18S_r were determined by the A260 measurement (Qubit 4 Fluorimeter by Invitrogen, California, USA), and the copy number was calculated based on the plasmid molecular weight (3929 bp). A series of tenfold dilution of plasmid incorporating the insert were prepared in sterile ddH_2_O. These serial dilutions were primarily used to determine the limit of detection (LOD) for each system.

Standard curves were constructed with the PCR results from three replicates per dilution, by plotting the crossing point values (Ct value) against the logarithm of the DNA copy number. Average values and standard deviation for the crossing point values were also calculated. The absolute copy number of the plasmid in relation to the starting amount of DNA was calculated accordingly to the formula: number of copies detected = amount of DNA(g) × 6.022 × 10^23^/fragment(bp) × 650.

### 2.8. Validation of 18S DNA as Internal Control

To evaluate the robustness of the Iberian hare 18S rRNA housekeeping gene regarding its resistance to degradation, we extracted samples from brain, skin, heart, lung, duodenum, kidney, liver, and genital samples, and preserved them in different conditions. qPCR tests were carried out immediately after extraction, two weeks after freezing at −20 °C (without nucleic acids protection solutions), and two weeks after incubation at room temperature (without nucleic acids protection solutions) to assess the resistance of the internal control DNA. All tests were performed in duplicates.

### 2.9. Clinical Samples Validation

To assess the sensitivity and specificity of the method for LeHV-5 DNA detection in field samples, several hare samples available in the National Reference Laboratory of Portugal (INIAV, I.P.) were used. These samples were first analyzed with a nested PCR system [11] using the NZYTaq II 2× Colourless Master Mix (NZYTech, Lisbon, Portugal) and subsequently tested with the duplex PCR for LeHV-5 gB and 18S rDNA, using the Multiplex PCR NZYTaq 2× Colourless Master Mix (NZYTech, Lisbon, Portugal).

## 3. Results

### 3.1. Specificity of the Method

The tests performed in silico and in vitro did not show amplification for any of the etiological agents tested (14 bacteria in silico and 3 bacteria in vitro, 3 parasites in silico and 4 parasites in vitro, and 16 viruses in silico and 13 viruses in vitro), demonstrated the specificity and robustness of the method (results not shown).

### 3.2. Efficiency and Sensitivity of the LeHV-5 Taqman System

**Figure 1:**
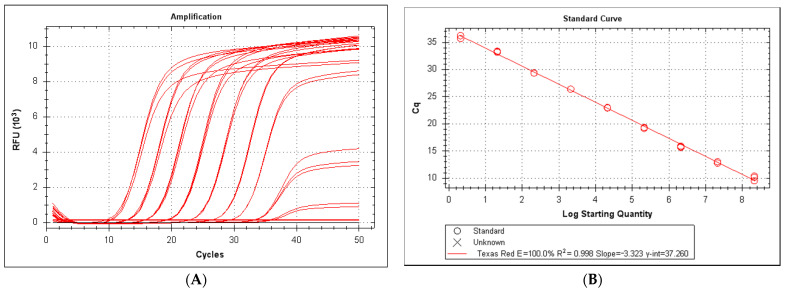
(**A**) Serial dilution from 2.1 × 10^8^ to 2.1 copies per reaction. Black lines correspond to negative Scheme 2. 1 × 10^−1^ and 2.1 × 10^−2^ copies per reaction). (**B**) Standard curve from 2.1 × 10^8^ to 2.1 copies per reaction.

A typical standard curve amplification plot and linear regression analysis for the LeHV-5 is shown in Figure 1. Excellent linearity was observed over nine orders of magnitude, from 2.2 × 10^8^ copies to 2.1 × 10^0^ copies (Figure 1A). The regression analysis for this interval yielded an R2 (correlation coefficient) of 0.998 and a y-intercept value of 37.26. The slope of 3.323 revealed a high qPCR efficiency (100.0%) (Figure 1B). The minimum number of copies of LeHV-5 DNA detected by the qPCR LeHV-5 gB system were 2.1 per 25 μL of reaction.

### 3.3. Efficiency and Sensitivity of the LeHV-5 in the EvaGreen System

**Figure 2:**
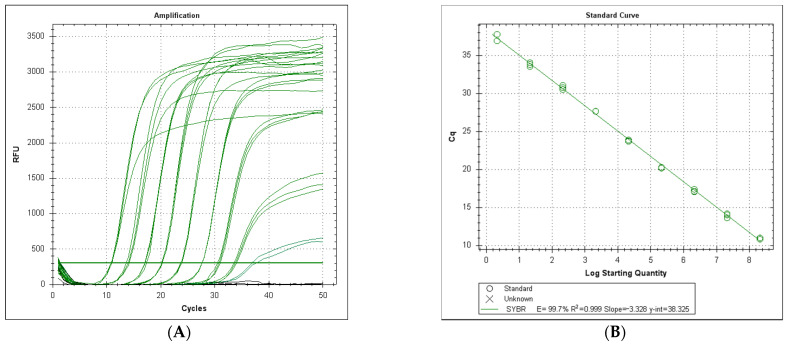

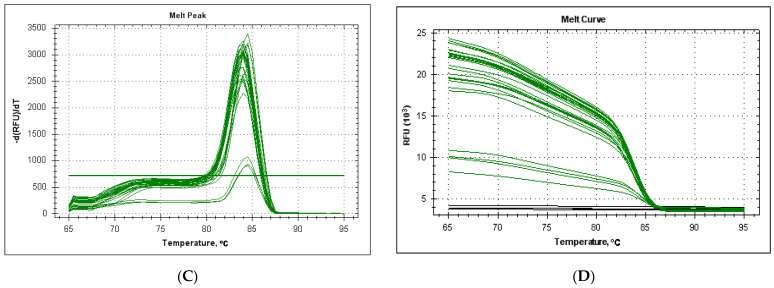
(**A**) Serial dilution from 2.1 × 10^8^ to 2.1 x 10^0^ copies per reaction. Black lines correspond to negative samples (2.1 × 10^−1^ and 2.1 × 10^−2^ copies per reaction). (**B**) Standard curve from 2.1 × 10^8^ to 2.1 × 10^0^ copies per reaction. (**C**) Melt point analysis for 36 reactions with 2.1 × 10^8^ to 2.1 × 10^0^ copies. (**D**) Melt curve analysis for 36 reactions with 2.1 × 10^8^ to 2.1 × 10^0^ copies.

A standard curve was generated by plotting the threshold cycles of reference standards versus their log copy number. Linear regression of the reference concentrations yielded a correlation coefficient of 0.999 (*p* < 0.001). The minimum number of copies detected by the system was 2.1 copies per 20 μL reaction for LeHV-5, with an amplification efficiency of 99.7% (Figure 2A,B).

As shown in Figure 2C, the melt curve analysis for the LeHV-5 gB amplifications showed a distinct unique peak in all reactions. The medium value for 36 analyses was 84.11 °C ± 0.21 °C. The variation in this temperature was more evident for the reactions with a lower number of DNA copies.

### 3.4. Behavior of 18S rDNA as an Internal Control

In silico analysis of the 18S rRNA gene showed that the designed system efficiently amplified a fragment of 117nt from Iberian hare and wild rabbit samples, as well as from several other mammalian species namely cat (*Felis catus),* harbor seal *(Phoca vitulina),* cheetah *(Acinonyx jubatus),* grizzly bear *(Ursus arctos horribilis),* Steller sea lion *(Eumetopias jubatus),* dingo *(Canis lupus dingo)*, dog *(Canis familiaris),* ref fox *(Vulpes vulpes)* among others, with which a minimum identity of 99.15% was observed, according to the nucleotide sequences currently available in public databases.

**Figure 3:**
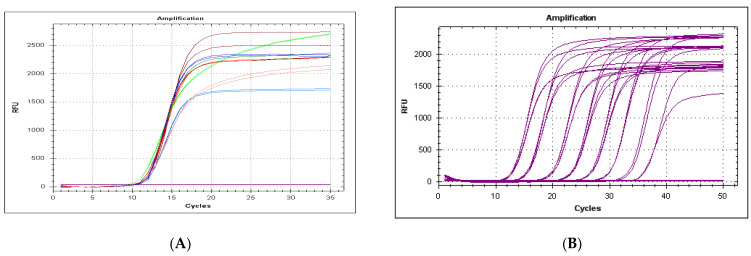

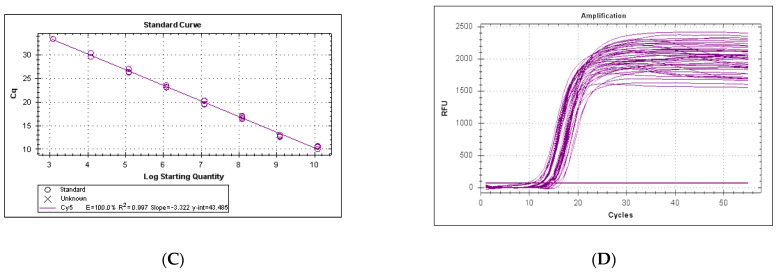
(**A**) Analysis of different primer concentrations for 18S DNA amplification. Fixed final concentration of the probe in all reactions (0.2 µM). Variable primer concentrations: 1 µM Fw primer, 1µM Rev primer (brown), 0.5 µM Fw primer, 1 µM Rv primer (dark green), 0.2 µM Fw primer, 1 µM Rev primer (light green), 1 µM Fw primer, 0.5 µM Rev primer (dark blue), 0.5 µM Fw primer, 0.5 µM Rv primer (red), 0.2 µM Fw primer, and 0.2 µM Rv primer (orange). (**B**) Serial dilution from 1.2 × 10^8^ to 1.2 × 10^1^ copies of p18S_ha per reaction. Black lines correspond to lack of amplification (1.2 × 10^0^ and 1.2 × 10^−1^ copies per reaction). (**C**) Standard curve from 1.2 × 10^8^ to 1.2 × 10^1^ copies per reaction. (**D**) Amplification of 40 samples of DNA (liver and spleen) from Iberian hare field samples.

The qPCR system for 18S amplification was robust when using different primer concentrations (Figure 3A). The minimum number of copies detected by the system was 12 copies per 25 μL reaction, with an amplification efficiency of 100% (Figure 3B,C).

The number of DNA copies detected in different field samples, homogenized at 20% (*w/v*) in PBS, was relatively consistent, ranging from Cq 9.16 to 16.24 (Figure 3D). When testing several samples without uniform homogenization (corresponding to 10–30% (*w/v*) final dilution in PBS), the 18S amplification Cq value ranged from 8.54 to 22.58.

To determine the robustness of the 18S qPCR, DNA was extracted from fresh hare samples and from the same samples after 7 days of incubation at 20–25 °C (RT) and at 37 °C. The Cq results are shown in Table 2, demonstrating that 18S rDNA was stable, even after one-week at 37 °C, showing a higher degradation in the liver, that could be justified by the parenchymal nature of this organ. The addition of antibiotic/antimycotic (AB/AM) had no obvious interference in the efficiency of DNA amplification, but the degree of liver putrefaction after 7 days was visible when compared to the Cq values obtained with the DNA extracted from a fresh liver sample. However, the putrefaction conditions tested had no relevant effect in the Cq values obtained in the lung and heart samples (Table 2).

### 3.5. Intra and Inter-Assay Reproducibility in the LeHV-5 Taqman and EvaGreen Systems

The reproducibility of the qPCR was evaluated with quadruplicates for each dilution replicated in two different assays. The Cq values for the intra-assay and inter-assay variability evaluations are shown in Table 3. A maximum coefficient of variation (CV) of 2.47% and 2.43% was obtained. These values, both under 3.5%, revealed a high repeatability and reproducibility. All Taqman assays were performed with a stable quantity of 1.2 × 10^6^ copies of p18S. In the case of EvaGreen, a singlexplex run was only carried out due to real-time differentiation limitations. However, a duplex using the EvaGreen system could be optimized and analyzed through high-resolution melt analysis.

### 3.6. Performance in the Diagnosis of Clinical Samples

The qPCR Taqman and EvaGreen systems allowed detection of a panel of positive samples, pre-diagnosed as LeHV-5 positive by the system described in [11], indicating a sensitivity of 100%. Likewise, both systems did not amplify any sample that was pre-classified as negative, showing a specificity of 100% (Table 4). All real-time PCR runs were validated by successful amplification of the 18S rRNA gene and sequencing.

Ten samples were negative in the qPCR, while a weak band of compatible size was generated in the nested PCR (Table 4). This band was sequenced and confirmed to be unspecific.

Contrarily, two doubtful bands in the nested PCR, which were positive in the qPCR were sequenced and confirmed to be gammaherpesvirus.

**Figure 4:**
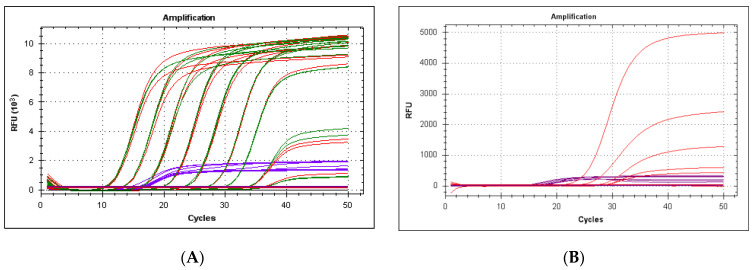
(**A**) Amplification of LeHV-5 gB gene in singleplex and duplex also for 18S rRNA gene (CY5 channel, purple curves). Red lines correspond to LeHV-5 amplification in singleplex reactions and green in duplex reactions. Horizontal black lines correspond to samples lacking amplification (2.1 × 10^−1^ and 2.1 × 10^−2^ copies per reaction) (**B**) Amplification of LeHV-5 (red curves) in duplex with the 18S rRNA gene (CY5 channel, purple curves), using field samples.

The performance of the qPCR as a duplex (LeHV-5 and 18S), evaluated by the Ct value and the fluorescence intensity, did not interfere with the efficiency of the LeHV-5 detection compared to the singleplex, either using both plasmids (Figure 4A, both efficiencies of 100%) or field samples (Figure 4B). Cross interaction between the two probes and channels was tested and excluded by analyzing the results obtained in the method described in Section 2.4. The value of Cq and relative fluorescence showed no significant interference when comparing singleplex and duplex assays, while using different probe concentrations. For this reason, we decided to use the minimum concentration of primers and probes recommended by the commercial kit, making the method cheaper.

## 4. Discussion

The Iberian hare is an iconic species of the Iberian Peninsula, which has a tremendous ecological, cultural, and economic importance. The direct or indirect impact of LeHV-5, a herpesvirus recently described in this species, on the present and future stability of the hare populations [9] is unknown, aggravated by the lack of census data.

Given the high prevalence of this virus in the samples analysed so far (46.7%, results not shown) and the shortness of knowledge on the disease it causes, the understanding of the true impact and dispersion of the LeHV-5 infection relies on a specific diagnostic method for monitoring and surveillance. The LeHV-5/18S rRNA gene duplex system developed and validated (described here), allows for the quantification of this virus in different tissue samples, and provides a diagnosis tool to perceive its tropism and physiopathology.

The sensitivity and specificity of this method were of 100% when using samples previously classified as positive or negative from our biobank, tested by a nested PCR for mammalian and avian herpesviruses [11]. Two samples classified as negative/doubtful by this nested PCR system, were successfully detected by the LeHV-5 qPCR. Ten samples were not detected by the LeHV-5/18S rRNA gene system, despite a compatible band being generated on the nested PCR, but sequencing was found to be interspecific. The gB gene was previously used as a target for several real-time PCR systems for other herpesviruses [12,13]. Using the standard curve generated by this method, researchers would be able to quantify the viral copies presented in the tissues analysed and verify the extraction and amplification success by the 18S rDNA behaviour.

To our knowledge, this represents the first method for the molecular diagnosis of LeHV-5, a virus first reported just some months ago [9].

## 5. Conclusions

In this article, we present the first rapid and quantitative diagnosis method for leporid gammaherpesvirus 5 (LeHV-5). This method proved to be robust, highly sensitive and highly specific, and could be used in two different formats, according to the laboratory preferences, namely by hydrolysis of a Taqman probe or green fluorescent nucleic acid dying (*EvaGreen*). Considering that the virus was reported for the first time in 2018, retrospective and prospective studies are important to elucidate the extension of the virus’ geographical spread in the hare populations. Finally, this study detected LeHV-5 in samples from 2017, providing evidence of the virus circulation before its first report in 2018.

## Figures and Tables

**Table 1:** Primers and probes designed for the qPCR systems.

*Gene Targeted*	Primers (Name/Nucleotide Sequence (5′–3′))	Probe (Name/Fluorophore/Sequence (5′–3′)/Quencher)
***gB LeHV-5***	**LeHV-5-gB Fw** GGACTCAGTGAACATTCACCAAAGCC **LeHV-5-gB Rv** CACCCACGATAAAAAAGTGCTCTGCC	**LeHV-5-gB_P** *[Texas Red]* TGCTCTCCAACACGCAGCTCGAAACATGCC *[BHQ1]*
***18S rRNA***	**18S Fw** TATGGTTCCTTTGGTCGCTCGCTC **18S Rv** TCTGATAAATGCACGCATCCCCCC	**18S Prb** *[CY5]* AGCTAATACATGCCGACGGGCGCTGACC *[BHQ2]*

**Table 2:** Cq values obtained with the 18S rDNA qPCR using European rabbit tissues submitted to different conservation/preservation conditions.

	Fresh			RT, 7 Days			37 °C, 7 Days		
Diluent	Liver	Lung	Heart	Liver	Lung	Heart	Liver	Lung	Heart
PBS	12.29	17.70	15.40	25.05	15.52	16.19	23.29	15.18	16.06
PBS, AB, AM	12.35	17.73	17.78	25.27	15.49	16.06	23.31	15.33	16.10

PBS (phosphate-buffered saline), AB—antibiotic, AM—antimycotic.

**Table 3:** Summary of statistical analyses of the repeatability and reproducibility results for both qPCR systems for gB detection.

**Copies/Reaction**	**Taqman System**					
	**Repeatability (Quadruplicates)**			**Repeatability (Three Independent Assays)**		
	Mean Cq	SD	%CV	Mean Cq	SD	%CV
2.1 × 10^8^	10.73	0.181	1.69	10.32	0.185	1.79
2.1 × 10^7^	13.44	0.093	0.69	13.55	0.101	0.75
2.1 × 10^6^	16.31	0.061	0.37	16.10	0.070	0.43
2.1 × 10^5^	20.03	0.086	0.43	19.86	0.084	0.42
2.1 × 10^4^	23.47	0.114	0.49	22.85	0.104	0.46
2.1 × 10^3^	27.31	0.080	0.29	27.44	0.082	0.30
2.1 × 10^2^	30.45	0.222	0.73	30.11	0.256	0.85
2.1 × 10^1^	34.07	0.159	0.47	34.05	0.175	0.51
2.1 × 10^0^	36.36	0.897	2.47	37.13	0.904	2.43
**EvaGreen SYSTEM**						
**Copies/reaction**	**Repeatability (Quadruplicates)**			**Repeatability (Three Independent Assays)**		
	Mean Cq	SD	%CV	Mean Cq	SD	%CV
2.1 × 10^8^	10.19	0.076	0.75	10.67	0.090	0.84
2.1 × 10^7^	13.31	0.074	0.56	13.80	0.114	0.83
2.1 × 10^6^	17.18	0.073	0.42	16.89	0.094	0.56
2.1 × 10^5^	20.70	0.188	0.91	20.14	0.250	1.24
2.1 × 10^4^	24.29	0.269	1.11	23.98	0.314	1.31
2.1 × 10^3^	27.91	0.044	0.16	27.50	0.098	0.36
2.1 × 10^2^	31.30	0.248	0.79	31.05	0.322	1.04
2.1 × 10^1^	34.89	0.177	0.51	34.60	0.198	0.57
2.1 × 10^0^	37.63	0.491	1.30	37.40	0.503	1.34

CV—coefficient of variation.

**Table 4:** Comparative final diagnosis between Nested PCR [11] and Taqman rt-qPCR system.

Sample Code	Nested PCR *		Real Time (Cq)	Estimated Copies/mg Tissue *
	Amplification	Sequencing Analysis		
**39189PT17**	N	-	NA	NA
**39190PT17**	P	LeHV-5	20.23	7.43 × 10^6^
**39191PT17**	N	-	NA	NA
**39192PT17**	P	LeHV-5	22.94	1.14 × 10^6^
**39344PT17**	P	LeHV-5	24.24	4.61 × 10^5^
**39351PT17**	N	-	NA	NA
**39355PT17**	N	-	NA	NA
**00751PT18**	N	-	NA	NA
**00807PT18**	N	-	NA	NA
**00813PT18**	P	LeHV-5	24.93	2.86 × 10^5^
**00814PT18**	P	LeHV-5	23.87	5.96 × 10^5^
**00815PT18**	P	LeHV-5	22.32	1.75 × 10^6^
**00816PT18**	P	LeHV-5	25.62	1.77 × 10^5^
**00817PT18**	P	LeHV-5	23.62	7.09 × 10^5^
**00818PT18**	N	-	NA	NA
**04971PT18**	N	-	NA	NA
**0492PT18**	N	-	NA	NA
**04483PT18**	N	-	NA	NA
**04985PT18**	N	-	NA	NA
**26935PT18**	N	-	NA	NA
**30901PT18**	N	-	NA	NA
**33014PT18**	D	unspecific	NA	NA
**33021PT18**	D	unspecific	NA	NA
**35866PT18**	D	unspecific	NA	NA
**35867PT18**	D	unspecific	NA	NA
**38457PT18**	N	-	NA	NA
**35869PT18**	P	LeHV-5	21.06	4.18 × 10^6^
**38455PT18**	P	LeHV-5	21.44	3.21 × 10^6^
**39375PT18**	D	LeHV-5	26.10	1.27 × 10^5^
**41434PT18**	D	unspecific	NA	NA
**41439PT18**	D	unspecific	NA	NA
**41440PT18**	D	unspecific	NA	NA
**00129FPT19**	D	unspecific	NA	NA
**03230PT19**	D	unspecific	NA	NA
**4033PT19**	P	LeHV-5	25.08	2.58 × 10^5^
**22059PT19**	P	LeHV-5	26.45	9.97 × 10^4^
**4037PT19**	P	LeHV-5	25.02	2.69 × 10^5^
**4064PT18**	P	LeHV-5	14.27	4.63 × 10^8^
**24980PT19**	D	unspecific	NA	NA
**29959PT18**	P	LeHV-5	25.19	2.39 × 10^5^
**1129PT18**	P	LeHV-5	23.65	6.94 × 10^5^
**29973PT18**	P	LeHV-5	25.71	1.67 × 10^5^
**30908PT18**	D	herpesvirus	28.36	2.65 × 10^4^
**33023PT18**	P	LeHV-5	22.57	1.47 × 10^6^
**30903PT18**	P	LeHV-5	26.59	9.05× 10^4^

P—positive, N—negative, D—doubtful. NA (no amplification). * Estimative using the standard curve of Taqman system.

## Data Availability

The data presented in this study are available on request from the corresponding author.

## Declaration

This study did not use live animals and was carried out within the scope of a National Plan for the Control of Rabbit Haemorrhagic Disease Virus 2 in rabbits (Dispatch no. 4757/2017 of 31 May), with the legal authorizations from the National Authority—The Institute for Nature Conservation and Forests (Instituto da Conservação da Natureza e das Florestas, ICNF, I.P.).
